# Genomic signature driving preinvasive to invasive processes in stage I lung adenocarcinoma

**DOI:** 10.1002/ijc.70282

**Published:** 2025-12-05

**Authors:** Biqin Mou, Yishan Duan, Jing Wang, Tiantian Li, Yuwei Huo, Xia Xiao, Conghui Cui, Zhujun Deng, Qiongxia Hu, Juan Jiang, Yiwei Liang, Sifen Lu, Xintong Tao, Kang Xie, Xinru Xiong, Niu Zhu, Liyun Bi, Faqiang Zhang, Weimin Li, Bojiang Chen

**Affiliations:** ^1^ Precision Medicine Center, Precision Medicine Research Center West China Hospital, Sichuan University Chengdu China; ^2^ Precision Medicine Key Laboratory of Sichuan Province West China Hospital, Sichuan University Chengdu China; ^3^ State Key Laboratory of Respiratory Health and Multimorbidity West China Hospital, Sichuan University Chengdu China; ^4^ Department of Respiratory and Critical Care Medicine West China Hospital of Sichuan University Chengdu Sichuan Province China

**Keywords:** genomic signature, invasive adenocarcinoma, lung adenocarcinoma, minimally invasive adenocarcinoma, prognosis

## Abstract

Progression from minimally invasive adenocarcinoma (MIA) to invasive adenocarcinoma (IA) in lung adenocarcinoma (LUAD) is associated with a significantly worse prognosis and lacks predictive markers. The genomic molecular mechanisms of progression and genetic signatures mediating the MIA to IA transition in early‐stage LUAD are still largely uncharacterized. In our study, a genomic signature driving MIA to IA was developed by 243 MIA and 532 IA stage I LUAD patients, and its ability to predict outcomes was validated in multiple cohorts. Among patients with stage I LUAD, 19 genes exhibited significant differences in frequency between MIA and IA groups, with notable enrichment in the MAPK, PI3K‐Akt and ErbB pathways. A genomic signature of 11 genes associated with LUAD invasion progression, with *TP53* and *CDKN2A* playing key functional roles, was developed and correlated with poor prognosis by internal and external cohorts (*p* < 0.05). The high‐risk group exhibited elevated tumor mutational burden, mutation‐allele tumor heterogeneity, and variant allele frequency values both in train and validation cohorts (*p* < 0.001). Mixed ground‐glass opacity and solid nodules, predominantly larger than 1 cm, were more common in the high‐risk population (*p* < 0.001), while the low‐risk group exhibited a higher proportion of high‐medium differentiated LUAD (*p* < 0.001). Our results reveal an 11‐gene genomic signature driving invasive progression from MIA to IA associated with poor outcome in stage I LUAD patients by validating internal and external cohorts, radiological, pathological and tumor size, with potential future implications for disease monitoring, prognosis, and future therapeutic interventions.

## Graphical abstract

What's new?

The progression of lung cancer from minimally invasive adenocarcinoma (MIA) to invasive adenocarcinoma (IA) is a complex process involving molecular and microenvironment changes. Key molecular events that drive the invasion process, however, remain poorly characterized. In this comparison of gene profiles and differentially mutated genes in MIA and IA, the authors identified a genomic signature associated with lung adenocarcinoma (LUAD) progression. In particular, the risk of poor prognosis was linked to tumor mutational burden, mutant‐allele tumor heterogeneity, and variant allele frequency. The findings offer a molecular foundation for risk stratification and personalized treatment strategies for the clinical management of LUAD.

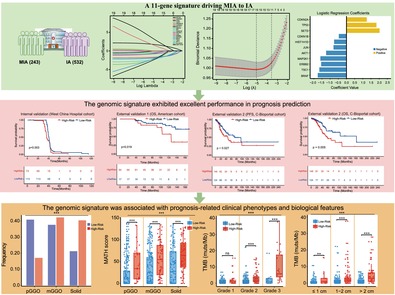

**Abbreviations**
AUC: area under the curve
CNVs: copy number variations
DCA: decision curve analysis
DFS: disease‐specific survival
FFPE: formalin‐fixed, paraffin‐embedded
IA: invasive adenocarcinoma
IGV: integrative genomics viewer
INDELs: small insertions and deletions
KEGG: Kyoto encyclopedia of genes and genomes
LASSO: least absolute shrinkage and selection operator
LUAD: lung adenocarcinoma
MATH: mutant‐allele tumor heterogeneity
mGGO: mixed ground‐glass opacity
MIA: minimally invasive adenocarcinoma
NGS: next‐generation sequencing
OS: overall survival
pGGO: pure ground glass opacity
PPI: protein–protein interaction
RNA: ribonucleic acid
ROC: receiver operating characteristic
SNVs: single nucleotide variants
TMB: tumor mutational burden
VAF: variant allele frequency
WES: whole‐exome sequencing

## 1 INTRODUCTION

Lung cancer is associated with the highest morbidity among malignant tumors and is the leading cause of cancer‐related deaths worldwide.^1^ CT‐guided lung cancer screening reduces mortality by 26%–61%, highlighting the importance of early detection to lower incidence and mortality.^2^ However, primary prevention trials have shown limited success, largely due to an incomplete understanding of early‐stage cancer development. Lung adenocarcinoma (LUAD), the most prevalent subtype, progresses through precursor lesions to minimally invasive adenocarcinoma (MIA), and eventually to invasive adenocarcinoma (IA). According to the 2021 WHO classification of lung tumors, MIA is defined as an isolated adenocarcinoma with a diameter of <30 mm accompanied by invasion of the basement membrane.^3^ Due to the limited invasive components of MIA, the estimated 10‐year postoperative disease‐specific survival (DFS) rates and overall survival (OS) rates are 100% and 97.8%, but when the tumor progresses to IA, the prognosis is significantly worse than that of MIA.^4^, ^5^ Therefore, exploring key molecular events that drive the invasion process from MIA to IA is promising for accurately identifying the subset of early‐stage lung cancer patients most likely to suffer from postoperative recurrence and mortality, thereby enabling timely intervention and improving prognosis.

The development of multiple omics analysis techniques, including transcriptomics, metabolomics, and genomics, has created opportunities to correlate tumor biology with clinical cancer phenotypes and elucidate the mechanism of tumor molecular evolution.^6^, ^7^ On the basis of these emerging omics data, contemporary investigations have characterized the genetic and immunological profiles of such lesions, elucidating key molecular drivers of LUAD pathogenesis.^8^, ^9^, ^10^ The evolutionary metabolic landscape from preneoplasia to invasive LUAD revealed that aberrant bile acid metabolism could be exploited for stratifying patients.^11^ In addition, a comparison of whole‐exome sequencing (WES) and ribonucleic acid (RNA) sequencing results from preinvasive and invasive samples revealed that tumor mutational burden (TMB), mutations in *TP53*, the APOBEC signature and arm, and focal copy number alterations were associated with increased genomic aberrations from the preinvasive to invasive stage and were strongly associated with invasiveness during tumor progression.^12^ However, the risk stratification reported in these studies mainly focused on differentially expressed genes in the transcriptome, significantly differential metabolites in metabolomics or clinical characteristics at various stages of lung cancer.^11^, ^12^, ^13^ The high cost, complexity of technology, and time consumption of transcriptomics and metabolomics testing have hindered their clinical application. In contrast, the increasing and widespread application of targeted next‐generation sequencing (NGS) to analyze tumor genomic profiles in clinical settings has provided the possibility to solve the urgent need for clinically available tools. The association between genomic alterations and prognosis has been verified by NGS analysis of 98 Chinese patients with advanced biliary tract cancer.^14^ However, the genetic mutation signatures underlying the invasiveness and prognosis from MIA to IA of stage I LUAD remain elusive.

Therefore, exploring tumor invasion‐related variables and validating their reliable associations with outcomes are key factors driving the development of precision oncology management for stage I LUAD. In this study, we compared gene profiles and differentially mutated genes between the MIA and IA cohorts to comprehensively explore the genomic events driving the progression from preinvasive to invasive adenocarcinoma based on targeted NGS techniques. Then, we constructed an 11‐gene genomic signature associated with poor prognosis and various clinical features such as tumor mutational burden, mutant‐allele tumor heterogeneity (MATH), and variant allele frequency (VAF), providing a comprehensive tool for risk stratification that could inform disease monitoring, prognosis prediction, and future therapeutic interventions, paving the way for personalized treatment strategies in LUAD. Furthermore, we integrated the genomic data with radiological, pathological and tumor size findings to enhance the potential clinical utility of the 11‐gene genomic signature, making it a valuable contribution to the understanding and management of LUAD.

## 2 MATERIALS AND METHODS

### 2.1 Data source

This retrospective cohort study included patients who received targeted NGS of 1021 cancer‐related genes (Table S1) at West China Hospital of Sichuan University from January 2021 to October 2023. Patient data for clinical characteristic validation were retrospectively collected from September 2023 to June 2024. The OS data used for internal validation were censored at the final follow‐up date of April 2025. The internal validation cohort and external validation cohorts from the cBioPortal cohort and another reported American cohort were used to evaluate the effectiveness of the genomic signatures constructed in our study as prognostic indicators.

The inclusion criteria for participants were as follows: (1) aged ≥18 years; (2) pathologically diagnosed with stage I LUAD after surgery (AJCC 8th Edition, 2017);^15^ (3) histologic subtypes (MIA and IA) were confirmed by two senior pathologists who underwent specific training; (4) patients volunteered for the 1021‐gene panel test, and genomic DNA was isolated from resected tumor tissue to identify genomic alterations; and (5) clinical, radiological, and histopathological information was available and traced through electronic medical records. The exclusion criteria were as follows: (1) patients with active tumors in other sites and (2) unqualified samples or gene test data.

### 2.2 Sample processing and DNA extraction

The genomic DNA (gDNA) in the formalin‐fixed, paraffin‐embedded (FFPE) tissue samples was extracted by using the QIAamp DNA FFPE Tissue & Blood Mini Kit (Qiagen, Hilden, Germany). The DNA concentrations in the tissue samples were measured using a Qubit fluorometer and a Qubit dsDNA HS (High Sensitivity) Assay Kit (Invitrogen, Carlsbad, CA, USA), whereas the DNA concentrations in PBL were measured using a Qubit 3.0 fluorometer and a Qubit dsDNA HS (High Sensitivity) Assay Kit (Thermo Fisher Scientific, Inc., Carlsbad, CA, USA).

### 2.3 Targeted NGS gene panel sequencing

Tumor tissue DNA was isolated using a QIAamp Circulating Nucleic Acid Kit (Qiagen) according to the manufacturer's instructions at the Precision Medicine Center, West China Hospital. Each DNA sample used for Qubit quantification was fragmented, and sheared DNA, approximately 170 bp in length, was used for end‐repair, A‐tailing, and targeted adapter ligation with unique identifiers, followed by amplification by polymerase chain reaction. Thereafter, all libraries were hybridized to a customized panel of 1021 cancer‐related genes including all the exon regions of 407 genes; the introns, promoters, or fusion breakpoints of 49 genes; and the coding regions of 611 genes (Table S1) at the Geneplus‐Beijing Institute (Beijing, China). DNA sequencing was performed using the Gene+Seq‐2000 sequencing system (GenePlus, Suzhou, China) per the manufacturer's guidelines.

### 2.4 Mutation analysis

The raw sequencing data were processed to remove terminal adaptor sequences and low‐quality reads via realSeq (version 3.1.0.20201208, in‐house) and NCfilter (version 2.0.0, in‐house). Clean reads were aligned to the human genome (GRCh37) via the Burrows–Wheeler Aligner (BWA, version 201808). GATK (version 201,808) was used to mark PCR duplicates, rematch the indel regions, and recalibrate the base mass value. Single nucleotide variants (SNVs) and small insertions and deletions (INDELs) were identified using realDcaller2 (version 2.0.0) and TNSCOPE (version 201808) and then annotated by somVASrealDcaller (version 1.101), somVASTNScope (version 1.0), and NCanno (version v.1.15). The in‐house software somMerge (version 1.0) was used to review hotspot variants and merge the results of these analyses. Copy number variations (CNVs) were called by CNVKIT (version 0.9.6) and annotated via annocnv (version 1.0, in‐house). An in‐house algorithm, NCsv2 (version 1.0.0), was used to identify split‐read and discordant read pairs to identify SVs, which were then annotated with annosv (version 1.0, in‐house). The sequencing coverage and quality statistics for each sample are summarized in Table S2. All samples should achieve median coverage (and range) per targeted base >200, with >80% targeted bases with coverage ≥200 (the threshold of some samples may be <80% based on actual clinical detection and other results). Four criteria were applied for competent mutations (SNVs, INDELs, and SVs): (1) somatic origin (excluding germline mutations); (2) mutation located in the coding region, nonsynonymous SNVs/indels, affecting ±2 splices; (3) VAF ≥0.7%; and (4) manual review of all candidate somatic mutations using the bioinformatics pipeline via the Integrative Genomics Viewer (IGV) through assessment of the quality of base calls, mapping quality of the reads, and overall read depth at each mutation site. For the CNVs, the results were filtered by combining the copy number (gain ≥3.4, loss ≤1.2) with a trusted copy number variation diagram.

### 2.5 Pathway and functional enrichment analysis

Kyoto Encyclopedia of Genes and Genomes (KEGG) enrichment analysis was performed via the ClusterProfiler package.^16^ Gene IDs were converted using the R package org.Hs.eg.db^17^ and the significance threshold of enrichment was set at *p* < 0.05. The enrichment results were visualized with the R packages enrichplot and ggplot2.^18^ Additionally, protein–protein interaction (PPI) networks were analyzed using the STRING database (https://stringdb.org, Version 11.0), which focuses on genes from different groups on the basis of mutation type.

### 2.6 TMB/VAF/MATH calculation

To minimize the influence of tumor content on TMB and VAF, samples of tumor cell content >20%, and the VAF corrected by recommended guidelines,^19^ were selected for TMB calculation, thereby improving the reliability of their application in tumor analysis. The TMB is calculated as the number of total nonsynonymous mutations divided by the length of the panel‐covered genomic region (muts/mb). VAF is defined as the proportion of mutated allele reads to the total number of reads at a given genomic site. The MATH score is calculated based on the percentage ratio of the median absolute deviation (MAD) to the median of the mutant‐allele fractions across the mutated genomic loci of the tumor, as follows: MATH = MAD/median × 100%.^20^

### 2.7 Genomic signature risk stratification construction and validation

Differentially mutated genes for intergroup comparisons were screened using the chi‐square test, followed by least absolute shrinkage and selection operator (LASSO) to screen out genes identified as most strongly associated with the progression of early LUAD. Variables with a p value <0.05 were selected to develop a logistic regression model. Finally, the performance of predictive accuracy in terms of discrimination and calibration was validated using the area under the curve (AUC) of the receiver operating characteristic (ROC) curve, calibration curve, and decision curve analysis (DCA) curves. Patients were stratified into high‐ and low‐risk groups according to the risk stratification value calculated from the model, and prognostic data from one internal and two external validation cohorts were used to assess the effectiveness of the signature as an independent prognostic parameter.

### 2.8 Co‐occurrence analysis of genomic signature and clinicopathological features

For clinical translation validation, the genomic prognostic signature was interrogated against four pivotal biomarkers: TMB, MATH, tumor size, and VAF. Additionally, cross‐domain data fusion was achieved by aligning the genomic risk signature with MRI‐based radiomics signatures, such as pure ground glass opacity (pGGO), mGGO, Solid, and histopathology features.

### 2.9 Statistical analysis

We have a quantitative estimate of the tumor purity for each sample analyzed using the ABSOLUTE algorithm (v1.6) with 95% confidence intervals.^9^ The quantitative data are presented as the means ± SD and were compared via either the independent samples *t*‐test or the Wilcoxon rank‐sum test. Categorical variables are expressed as counts and percentages (%), and differences between cohorts were compared via the chi‐square test or Fisher's exact test, as appropriate. Survival curves were generated via the Kaplan–Meier method, and the log‐rank test was performed to evaluate the significance of differences. All comparisons were analyzed using two‐tailed tests, with statistical significance set at *p* < 0.05. Statistical analyses were performed using R (V.3.6.1) software.

## 3 RESULTS

### 3.1 Patient characteristics

**FIGURE 1:**
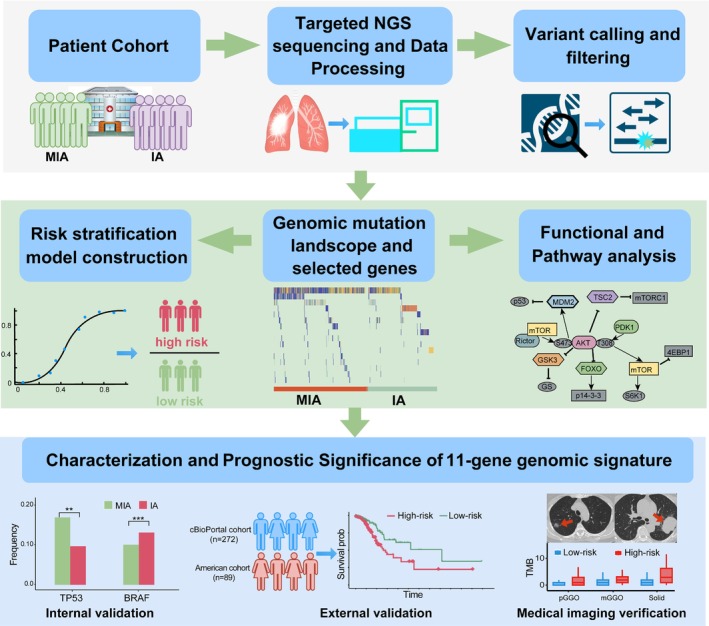
Workflow of the study and characterization of the 11‐gene genomic signature in early‐stage LUAD. The process includes patient cohort selection, targeted NGS sequencing, variant filtering, and construction of a risk stratification model. Genomic mutations and functional pathways are analyzed, followed by validation of the 11‐gene signature's prognostic value, showing significant associations with survival, tumor characteristics, and mutation patterns between high‐risk and low‐risk groups.

A total of 775 patients with stage I LUAD, consisting of 243 MIA and 532 IA patients, were recruited for our research. The clinical, radiological and pathological data are summarized in Table 1. Moreover, patient characteristics, gene data, and prognosis information for the internal (clinical characteristic, *n* = 348; OS, *n* = 186) and external validation cohort (cBioPortal cohort, *n* = 272; American cohort, *n* = 89) were obtained. The analysis process of this study is outlined in Figure 1.

**TABLE 1:** Baseline patient characteristics comparison between the MIA and IA cohort.

Parameters	MIA (*n* = 243)	IA (*n* = 532)	*p* value
*Clinical information*			
Age	46.0 ± 10.5	58.8 ± 11.1	<0.001
Gender			<0.001
Female	184/243 (75.7%)	334/532 (62.8%)	
Male	59/243 (24.3%)	198/532 (37.2%)	
History of smoking	24/243 (10.1%)	100/532 (18.8%)	0.002
Family history of cancer	36/243 (15.2%)	86/532 (16.2%)	0.672
*Radiological information*			
Nodule diameter (mm)			<0.001
≤10	190/238 (79.8%)	64/512 (12.5%)	
10–20	45/238 (18.9%)	257/512 (50.2%)	
>20	3/238 (1.3%)	191/512 (37.3%)	
Nodule density			<0.001
pGGO, pure ground‐glass opacity	173/238 (72.7%)	95/512 (18.6%)	
mGGO, mixed ground‐glass opacity	55/238 (23.1%)	235/512 (45.9%)	
Solid	10/238 (4.2%)	182/512 (35.5%)	
*Pathological information*			
Histological grade			
Grade 1, low differentiated	NA	149/455 (32.7%)	NA
Grade 2, moderately differentiated	NA	261/455 (57.4%)	NA
Grade 3, highly differentiated	NA	45/455 (9.9%)	NA

*Note*: NA: indicates data is not available.

As summarized in Table 1, a higher proportion of older male patients with a history of smoking was observed in the IA cohort. Not surprisingly, the nodules in the IA cohort were larger in diameter, and a greater proportion of them were mixed ground‐glass opacity (mGGO) and solid nodules.

### 3.2 Genomic landscape and pathological progression driven by different genomic events

**FIGURE 2:**
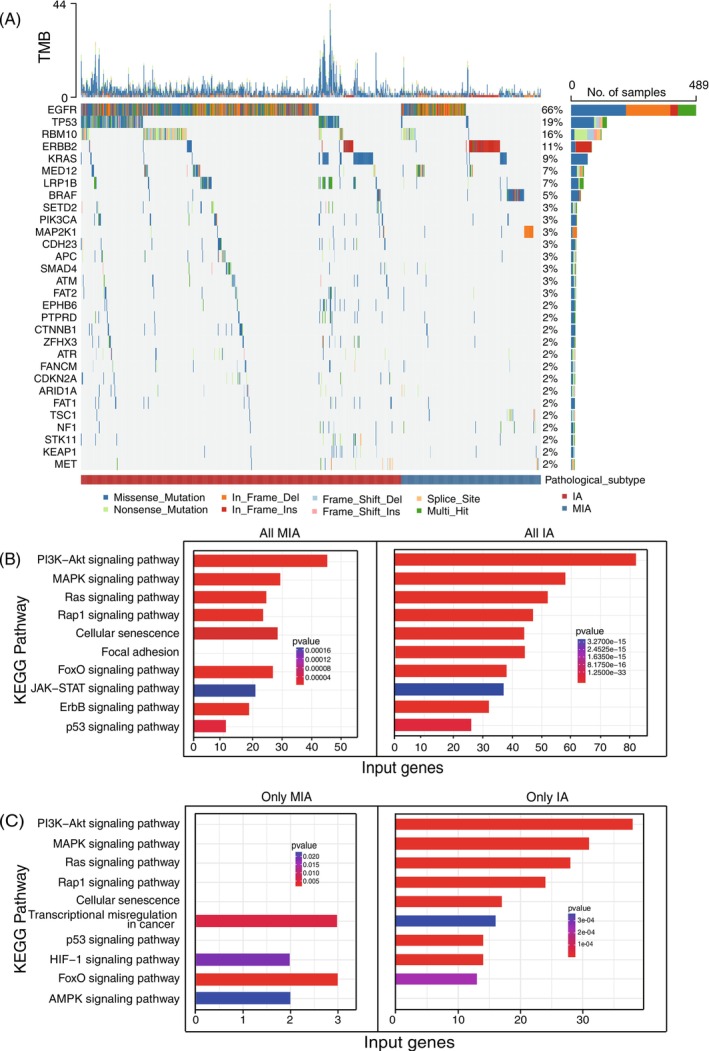
Genomic characteristics of MIA and IA. (A) Waterfall plots showing the frequency and types of mutations found in the TOP30 mutated genes in all patients. (B) KEGG pathway enrichment analysis of all mutated genes in IA and MIA. (C) KEGG pathway enrichment analysis of mutated genes unique to IA and MIA.

All quality controls in our NGS analysis met the acceptance criteria, and the detailed results could be found in Table S2. As shown in Figure 2A, mutation profile analysis roughly distinguished MIA from IA. The overwhelming majority of patients (93.68%, 726/775) harbored gene mutations. As the most frequently mutated gene in both MIA and IA, *EGFR* was mutated in as high as 66% of patients. The second most frequent mutation is *TP53*, with a frequency of 19%. Among the MIA samples, *EGFR* (46%), *ERBB2* (23%), and *BRAF* (13%) were significantly mutated (Figure S1A). In contrast, the three genes most frequently mutated in IA patients were *EGFR* (74%), *TP53* (26%), and *RBM10* (19%) (Figure S1C). The mutation frequencies of *ERBB2* and *BRAF* mutations in MIA patients tended to reduce the malignancy of cancer significantly more than those in IA patients, whereas *TP53* and *CDKN2A* mutations that increased cancer malignancy were more prevalent in the IA group. Furthermore, we observed that the co‐mutation of *EGFR* and *TP53* was significantly higher in IAs than in MIAs (Figures 2A and S1A,C). Additionally, the observed variant classification and types and SNVs in the MIA cohort were broadly similar to those observed in the IA cohort. Compared with IA, MIA exhibited a lower number of mutated genes and lower mutation frequencies (Figure S1B,D). Most genomic alterations found in IA were already present in MIA (Figure S2A).

Compared with MIA patients, the IA cohort exhibited a higher number of mutations, as well as TMB, MATH and VAF levels (Figure S2C–F). The combined effects of tumor heterogeneity and mutation frequency showed minimal differences between MIA and IA (Figure S2B). Our findings indicated that patients with solid nodules or larger lesion diameters in the IA subgroup had significantly higher TMB, MATH and VAF scores than those in the MIA subgroup (Figure S3A,B). In addition, the differentiated pathological stage of IA was also significantly associated with TMB, MATH and VAF (Figure S3C).

### 3.3 Enrichment analysis of the invasive‐associated genes

KEGG pathway enrichment analyses revealed that mutated genes in both MIA and IA, as well as those unique to IA, were enriched in similar signaling pathways, including the classical PI3K‐Akt signaling pathway, MAPK signaling pathway, Ras signaling pathway and Rap 1 signaling pathway (Figure 2B). What sets IA apart from MIA was that IA samples had more mutations in genes involved in focal adhesion signaling (Figure 2B). Another distinct signal pathway that drives the pathological process of IA and MIA is the AMPK signal pathway (Figure 2C), which is enriched in mutated genes unique to MIA.

### 3.4 Key gene screening and risk stratification model construction

**FIGURE 3:**
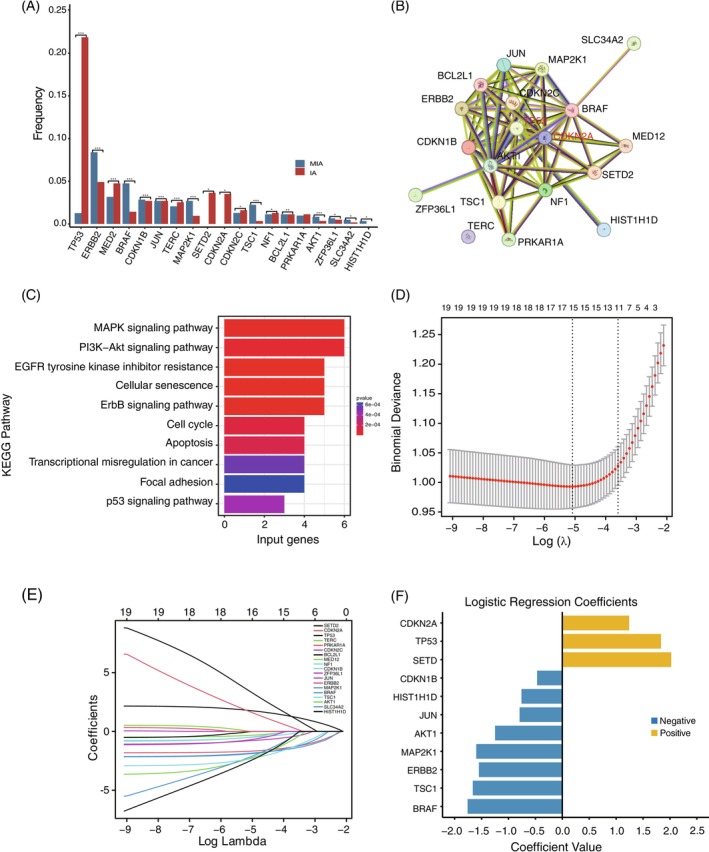
Screening of key genes driving preinvasive to invasive lung adenocarcinoma progression to construct predictive models. (A) 19 genes with significantly different mutation frequencies between the MIA and IA cohorts were screened. (B) Protein–Protein Interaction Networks (PPI) of 19 differential mutated genes identified *TP53* and *CDKN2A* as the key genes associated with disease progression in LUAD. (C) KEGG pathway enrichment analysis of 19 differently expressed genes between IA and MIA cohorts. (D) The binomial deviation loss function identified 11 genes as the optimal number of genes included in the logistic regression model. (E) Least absolute shrinkage was performed with the minimum criteria. (F) Coefficient values of mutated genes that were significantly associated with disease progression in early LUAD in the logistic regression model.

Our results revealed that mutations in 19 genes were significantly different in frequency between MIA and IA, which might be involved in the progression from MIA to IA (Figure 3A). In the 19 significantly different mutated genes, *TP53* and *CDKN2A* are presumed to be key in driving the pre‐invasive to invasive progression in stage I LUAD according to protein–protein interaction (PPI) networks (Figure 3B). Not surprisingly, differently mutated genes between MIA and IA were predominantly involved in the activation of oncogenic pathways, including the MAPK and PI3K‐Akt networks indicated by the KEGG database which also associated with tumor invasion (Figure 3C).

Based on the LASSO regression model, 11 mutated genes most closely associated with invasion progression in stage I LUAD were selected for risk stratification model construction (Figure 3D,E), and the ranks and coefficient values of these 11 genes in the LR model are shown in Figure 3F. Figure S4 shows the discriminative performance of the LR model in terms of ROC curves (AUC = 0.78), and the LR model shows a good predictive effect in risk stratification for early lung cancer invasion. Moreover, the LR model was verified to exhibit favorable clinical utility according to the calibration curves and DCA curves. The LR model had the best discrimination ability, with the accuracy (0.80), specificity (0.92), NPV (0.82) and PPV (0.70).

The risk stratification value of each sample was calculated using the following formula:

Value = 1.83331403*TP53 + 1.24152921*CDKN2A + 2.02331501*SETD2 − 1.54969391*ERBB2 − 1.75892268*BRAF − 1.66240923*TSC1 − 1.59870732*MAP2K1 – 0.46874594*CDKN1B − 0.79330108*JUN − 1.24918034*AKT1 – 0.75809038*HIST1H1D, where the gene name indicates whether the gene is detected, with a value of 0 or 1.

### 3.5 Prognostic significance of the 11‐gene genomic signature risk stratification via multiple cohorts

**FIGURE 4:**
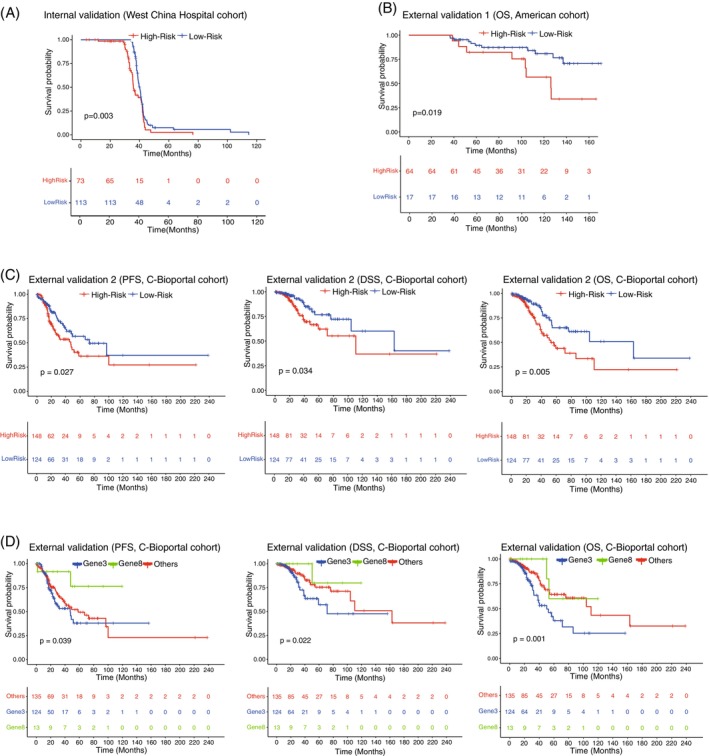
Prognostic assessment of high‐risk and low‐risk subtypes according to risk stratification of the logistic regression model enrolled 11‐gene genomic signature. (A) Patients with high‐risk genomic signature in the internal cohort had worse OS than those with a low‐risk signature. (B) Patients with high‐risk genomic signature in the American cohort had worse OS than those with a low‐risk signature. (C) Patients with a high‐risk genomic signature in the cBioPortal database had worse PFS, DSS, and OS than the low‐risk population. (D) Tumors harboring mutations in any of the Gene8 sets (*CDKN1B*, *HIST1H1D*, *JUN*, *AKT1*, *MAP2K1*, *ERBB2*, *TSC1*, and *BRAF*) were associated with better PFS, DSS, and OS than those in the Gene3 sets (*CDKN2A*, *TP53*, and *SETD2*) and the rest of the population.

Patients were divided into high‐risk and low‐risk subtypes for tumor invasion according to the median value in the logistic regression model. As expected, patients in the high‐risk group showed poorer OS in our internal cohorts (Figure 4A). Patients with a high‐risk genomic signature in the American cohort had an inferior OS rate (*p* = 0.019) (Figure 4B). Similarly, patients in the low‐risk group in the cBioPortal database had significantly longer PFS (*p* = 0.027), OS (*p* = 0.005) and DSS (*p* = 0.034) than those in the high‐risk group (Figure 4C), which indicated that the prognostic significance of the 11‐gene genomic signature was verifiable.

In order of coefficient value, mutations in the Gene3 set, including *CDKN2A*, *TP53*, and *SETD2*, increased in frequency in the aggressive stage, which was well proven to have unfavorable PFS (*p* = 0.039), DSS (*p* = 0.022) and OS (*p* = 0.001) in further Kaplan–Meier survival analysis. Accordingly, populations with mutations in the Gene8 set, including *CDKN1B*, *HIST1H1D*, *JUN*, *AKT1*, *MAP2K1*, *ERBB2*, *TSC1*, and *BRAF*, which were much more frequently mutated in MIA samples than in the IA samples and exhibited a cancer suppressor role, had a significantly better prognosis compared with others (Figure 4D).

### 3.6 Characterization of the 11‐gene genomic signature risk stratification

**FIGURE 5:**
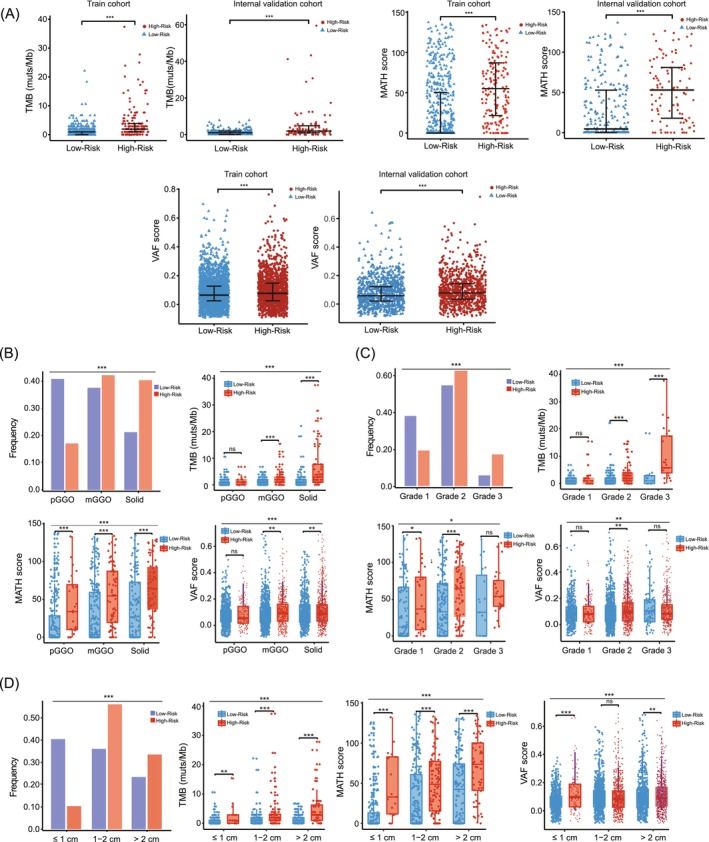
Characterization of the 11‐gene genomic signature risk stratification in the logistic regression model. (A) Higher TMB, MATH score and VAF score were observed among patients with a high‐risk genomic signature both in the train and validation cohorts. (B) The association between each of the three radiology subtypes of LUAD and genomic signature risk stratification according to the logistic regression model. (C) The association between pathological progression of LUAD and genomic signature risk stratification according to the logistic regression model. (D) The association between lesion diameter and genomic signature risk stratification according to the logistic regression model. *p < 0.05; **p < 0.01; ***p < 0.001.

Given the satisfactory prognostic performance of the 11‐gene genomic signature in patients with stage I LUAD, we further explored the underlying mechanism and its potential clinical utility. With respect to the biological characteristics obtained by targeted NGS, high‐risk populations exhibited higher TMB, MATH, and VAF levels than other populations both in train and internal validation cohorts (Figure 5A). A comparison of the radiological findings revealed that mGGO (41.5% vs. 38.2%, *p* < 0.005) and solid nodules (42.5% vs. 22.7%, *p* < 0.005) accounted for a higher percentage in the high‐risk population than in the low‐risk group and that the majority of the lesion diameters of the nodules were larger than 1 cm (89.6% vs. 62.2%, *p* < 0.005) (Figure 5B,D). The degree of tumor differentiation varied between the low‐risk and high‐risk cohorts, and the proportion of high‐medium differentiated LUAD was higher in the low‐risk subgroup (92.6% vs. 80.0%, *p* < 0.005) (Figure 5C). Notably, even when we accounted for the radiology subtype, lesion diameter, and degree of pathological differentiation, differences in the TMB, MATH and VAF scores between the high‐risk and low‐risk subtypes still persisted (*p* < 0.005) (Figure 5B–D). Detailed analysis of each gene enrolled in the 11‐gene signature was subsequently performed. *TP53*, *CDKN2A*, and *SETD2* in the Gene3 set were further shown to be mutated more frequently in patients with poor radiological presentation (including lesions with a more solid composition and larger diameter) and less pathological differentiation (Figure S5A–C). *ERBB2*, *BRAF* and *CDKN1B* in the Gene8 set, which act as tumor suppressors in early‐stage LUAD, were closely related to better radiological manifestations (Figure S5D–F).

## 4 DISCUSSION

This study established an 11‐gene signature integrating multi‐omics genomic features to enhance detection of early LUAD, demonstrating high sensitivity in distinguishing MIA from IA subtypes. Notably, this study constitutes the largest targeted sequencing analysis to date elucidating early LUAD progression from MIA to IA. Mutation profiles of stage I LUAD samples and the subgroups of MIA versus IA were established, and differential mutation genes between MIA and IA were identified. The 11‐gene signature characterizing the invasive process from MIA to IA was constructed and validated to serve as an effective prognostic biomarker not only validated by internal and external datasets, but also through assessment of pathology, radiological progress and tumor size, including TMB, MATH and VAF, as well as molecular characteristics relevant to early‐stage tumor invasion.

In our study, the detection rate of *EGFR* was as high as 66%, which was higher than the 49.1% reported in other Asian populations.^21^ The higher detection rate of *EGFR* may be due to the following reasons: Firstly, samples in this study were primarily obtained from western China, where people shared certain differences in lifestyle and genetic background.^22^ Secondly, our samples are derived from stage I LUAD tissues, mainly surgical specimens, with a tumor content of over 20%.^23^, ^24^ Additionally, the proportion of females or non‐smokers in this study was relatively high.^25^ Finally, we used a large panel, which includes exons and some introns of the major driver genes of patients with LUAD.^26^ Our analysis revealed that MIA samples harboured significant mutations in *EGFR*, *ERBB2* and *BRAF*, whereas IA patients were characterized by significant mutations in *EGFR*, *TP53*, and *RBM10*, which have previously been reported as commonly mutated in LUAD.^27^ Most genomic alterations found in IA were already present in MIA, which was also observed in other studies on the progression of early LUAD.^28^ Consistent with previous studies, no significant difference in the frequency of *EGFR* mutations between MIA and IA samples was observed, suggesting that driver mutations occur at early stages.^13^

KEGG pathway enrichment analyses revealed that besides those unique to MIA, mutated genes were enriched in similar signaling pathways associated with tumor progression and invasion, including the classical PI3K‐Akt, the MAPK and Ras signaling pathway.^29^, ^30^ IA samples exhibit more mutated genes in focal adhesion signaling, which correlated with tumor progression, metastasis and treatment by influencing cancer cells as well as stromal cells in the tumor microenvironment.^31^, ^32^ The AMPK signaling pathway that drives the pathological process of IA and MIA is enriched in mutated genes unique to MIA likely exhibits antitumor activity by regulating metabolism and inhibiting inflammatory reaction.^33^

To understand the key genetic mutation events in the progression of early LUAD, we identified 19 differential mutation genes that might be involved in the progression from MIA to IA of LUAD. Interestingly, *TP53*, *ERBB2* and *BRAF* were shared by both groups, suggesting that these mutations are early events that may play a progressive role in malignant transition, which is inconsistent with the results of other studies of *EGFR*, *TP53*, and *KRAS*.^9^, ^28^ Our study also identified *TP53* as presumed to be a key mediator in the invasive progression of LUAD. Compared with IA, MIA is closely associated with a lower frequency of *TP53* mutations, which is in line with the low frequency of *TP53* mutations in LUAD precursors.^9^ As a tumor suppressor gene that is frequently mutated in human cancers, *TP53* not only can promote tumor proliferation, metastasis and invasion through multiple mechanisms, including accelerating the acquisition of other driver mutations and causing extensive genomic damage due to defective DNA repair,^34^, ^35^ but also contributes to regulate tumor metabolism.^36^, ^37^, ^38^ *TP53* mutations are also closely related to abnormal cell cycle proliferation and concurrently suppress apoptosis regulators.^39^, ^40^ Through TGF‐β/Smad3‐dependent signaling, *TP53*‐mutant tumor cells activate cancer‐associated fibroblasts, inducing extracellular matrix remodeling and forming a pro‐metastasis microenvironment.^41^ Furthermore, our analysis revealed significantly higher proportions of smokers, mGGO and solid nodules in the IA cohort, which may potentially contribute to the elevated *TP53* mutation rate observed in these patients. Tobacco smoking can directly induce misreplication of DNA damage, including G>T transversion (SBS4[G>T]), which is a known cause of *TP53* mutations.^27^, ^42^ mGGO and solid nodules are clinically observed to exhibit greater aggressiveness and higher chromosomal instability, which may also explain the higher frequency of *TP53* mutations in these lesions.^12^, ^43^ Additionally, the survival prognosis of LUAD patients was related to *TP53* mutations and the presence of *TP53* mutations was associated with shorter OS in patients with LUAD.^44^

Genomic data was expected to facilitate risk‐stratified disease management for early‐stage LUAD, dramatically improving patient survival. Signatures based on gene expression profiles from RNA sequencing and microarray data have been derived for the prognosis of patients with LUAD alone and for the prediction of recurrence.^45^, ^46^, ^47^ However, rare clinical application and high cost of RNA sequencing and microarray have limited the clinical value of their derived gene signature. By contrast, the increasingly wide application of targeted NGS to analyze the genomic profiles of tumors in clinical settings has provided the possibility to solve the urgent need for clinical application. Therefore, we established an 11‐gene signature identified from a set of 19 differentially mutated genes between MIA and IA and confirmed its excellent performance in predicting patient prognosis trends not only by the ROC curve, calibration and DCA curves, but also across different datasets in stage I LUAD. Given the satisfactory prognostic performance of the 11‐gene genomic signature in patients with stage I LUAD, we further explored its underlying mechanism underlying its accurate prediction of clinical outcomes. High‐risk populations identified by the 11‐gene signature exhibited higher TMB, MATH, and VAF levels, which were reported with tumor invasion and poor prognosis. Particularly, TMB estimated by targeted NGS successfully improved the stratification of immunotherapy response in a study at Memorial Sloan Kettering Cancer Center.^49^ Moreover, the 11‐gene signature showed strong correlations with both radiological progression and the pathological differentiation level of the tumor lesion. Individual analysis of the 11 genes revealed that *TP53*, *CDKN2A*, and *SETD2* were associated with adverse features (poor radiology, low differentiation). In contrast, the *ERBB2*, *BRAF*, and *CDKN1B* were linked to more favorable radiological presentations in early‐stage LUAD.^48^, ^49^ The above features of the gene signature account for its link to tumor progression and its ability to accurately predict outcomes. Since the 11‐gene signature correlates with poor outcomes in these patients, the prognostic signature can aid in early diagnosis and risk assessment, helping clinicians identify high‐risk individuals who are more likely to progress from MIA to IA. Incorporating this genomic signature with radiological and pathological features could enhance early diagnostic accuracy and improve prognostic assessments for radiologists and pathologists. This enables earlier intervention, in which high‐risk patients may benefit from intensified surveillance and treatments such as targeted therapy, immunological therapy, or chemotherapy to prevent or delay disease progression, while low‐risk patients can avoid unnecessary aggressive treatments.

Several limitations constrain the interpretation of our findings. Chief among these is the absence of preinvasive lesions, especially atypical adenomatous hyperplasia (AAH) and adenocarcinoma in situ (AIS) in our cohort. The prevailing hypothesis posits pathological progression proceeds through the sequence from AAH, AIS to MIA and then to IA, so this absence substantially constrains our ability to investigate the molecular mechanisms underlying the transition from preinvasive to IA. As a result, we are unable to characterize early genetic and epigenetic alterations that drive tumor initiation and malignant progression, which represents a critical shortfall in elucidating the complete evolutionary trajectory of lung adenocarcinoma.^11^, ^28^ Future studies incorporating well‐annotated AAH/AIS samples and longitudinal multi‐omics analyses will be essential to fully elucidate the mechanistic continuum of LUAD development. Furthermore, the specific biological functions and molecular mechanisms of the key modules have yet to be fully elucidated, and future studies are needed to investigate their precise roles such as particularly MAPK, PI3K‐Akt, and ErbB signaling in the pathogenesis and progression of stage I LUAD. Additionally, our study did not incorporate multi‐region tumor samples thereby limiting our ability to characterize intratumoral spatial heterogeneity patterns that may influence tumor invasion. A key clinical challenge lies in conducting multi‐region NGS analysis within the same patient. Finally, our targeted sequencing approach focuses on 1021 cancer‐related genes, thereby providing less comprehensive genomic coverage of LUAD invasion than whole‐genome or exome methods would afford. Future investigations should incorporate whole exome/genome sequencing and larger micro‐dissected cohorts to better characterize invasiveness acquisition. Crucially, accurate identification of genes or evolutionary mechanisms underlying the early LUAD invasion process requires rigorous controlling of all potential confounding factors, for example, smoking history, gender.

## 5 CONCLUSION

In addition to detecting common driver gene mutations and guiding targeted therapies, targeted NGS further revealed prominent gene mutation profiles and pathways linked to stage I LUAD survival, providing a molecular basis for precision intervention. By screening differentially mutated genes from MIA/IA cohorts, we constructed an 11‐gene genomic signature for stage I LUAD progression. This signature demonstrated excellent prognostic performance with direct clinical utility, showing significant associations with prognosis‐related clinical phenotypes and biological features. Crucially, its ability to identify high‐risk recurrence/death populations in surgically resected stage I LUAD offers immediate value for postoperative management decisions.

## AUTHOR CONTRIBUTIONS

**Biqin Mou:** Data curation; software; writing – review and editing; writing – original draft; formal analysis; visualization; methodology. **Yishan Duan:** Data curation; formal analysis; visualization; writing – original draft; writing – review and editing; investigation. **Jing Wang:** Investigation; data curation; writing – review and editing. **Tiantian Li:** Investigation; data curation; writing – review and editing. **Yuwei Huo:** Investigation; data curation; writing – review and editing. **Xia Xiao:** Investigation; validation; writing – review and editing. **Conghui Cui:** Investigation; writing – review and editing. **Zhujun Deng:** Investigation; validation; writing – review and editing. **Qiongxia Hu:** Investigation; writing – review and editing. **Juan Jiang:** Investigation; writing – review and editing. **Yiwei Liang:** Investigation; writing – review and editing. **Sifen Lu:** Investigation; writing – review and editing. **Xintong Tao:** Investigation; writing – review and editing. **Kang Xie:** Investigation; writing – review and editing. **Xinru Xiong:** Investigation; writing – review and editing. **Niu Zhu:** Investigation; writing – review and editing. **Liyun Bi:** Investigation; writing – review and editing. **Faqiang Zhang:** Investigation; writing – review and editing. **Weimin Li:** Conceptualization; resources; data curation; supervision; funding acquisition; project administration; writing – review and editing. **Bojiang Chen:** Funding acquisition; conceptualization; resources; data curation; writing – review and editing; project administration; supervision.

## FUNDING INFORMATION

This project was supported by the National Natural Science Foundation of China (82573349), Noncommunicable Chronic Diseases‐National Science and Technology Major Project from the National Health Commission of China (2023ZD0506105/2023ZD0506100), Outstanding Youth Science Fund Project of Sichuan Natural Science Foundation (2024NSFJQ0051) and Major Research Program of the National Natural Science Foundation of China (92159302).

## CONFLICT OF INTEREST STATEMENT

The authors declare that they have no known competing financial interests or personal relationships that could have appeared to influence the work reported in this paper.

## ETHICS STATEMENT

All samples were collected with informed consent following the approval of the institutional review board of West China Hospital of Sichuan University (Ethics Approval No. 2024 [1903]) and the study was conducted in accordance with the Declaration of Helsinki and the International Conference on Harmonization Good Clinical Practice guidelines.

## Supporting information


**TABLE S1.** Gene list used in the 1021 gene panel testing.

**TABLE S2.** Sequencing coverage and quality statistics of all samples used in this study.

**FIGURE S1.** Gene mutation profiling of MIA and IA.
**FIGURE S2.** Genomic events which may drive MIA to IA.
**FIGURE S3.** Comparison of TMB, MATH score, and VA F scores between the IA and MIA cohorts associated with radiology, pathological and lesion diameter.
**FIGURE S4.** Evaluation of the 11‐gene prognostic signature.
**FIGURE S5.** Characterization of screened genes included in the risk stratification of the logistic regression model.

## Data Availability

R code is publicly available on GitHub (https://github.com/YanCCscu/GenomicSignatureLUAD). Other data that support the findings of this study are available from the corresponding author upon request.

## References

[1] Bray F , Laversanne M , Sung H , et al. Global cancer statistics 2022: GLOBOCAN estimates of incidence and mortality worldwide for 36 cancers in 185 countries. CA Cancer J Clin. 2024;74:229‐263. 38572751 10.3322/caac.21834

[2] de Koning HJ , van der Aalst CM , de Jong PA , et al. Reduced lung‐cancer mortality with volume CT screening in a randomized trial. N Engl J Med. 2020;382:503‐513. 31995683 10.1056/NEJMoa1911793

[3] Nicholson AG , Tsao MS , Beasley MB , et al. The 2021 WHO classification of lung tumors: impact of advances since 2015. J Thorac Oncol. 2022;17:362‐387. 34808341 10.1016/j.jtho.2021.11.003

[4] Nakao M , Yoshida J , Goto K , et al. Long‐term outcomes of 50 cases of limited‐resection trial for pulmonary ground‐glass opacity nodules. J Thorac Oncol. 2012;7:1563‐1566. 22878750 10.1097/JTO.0b013e3182641b5c

[5] Yotsukura M , Asamura H , Motoi N , et al. Long‐term prognosis of patients with resected adenocarcinoma in situ and minimally invasive adenocarcinoma of the lung. J Thorac Oncol. 2021;16:1312‐1320. 33915249 10.1016/j.jtho.2021.04.007

[6] Karjalainen MK , Karthikeyan S , Oliver‐Williams C , et al. Genome‐wide characterization of circulating metabolic biomarkers. Nature. 2024;628:130‐138. 38448586 10.1038/s41586-024-07148-y PMC10990933

[7] Li X , Quick C , Zhou H , et al. Powerful, scalable and resource‐efficient meta‐analysis of rare variant associations in large whole genome sequencing studies. Nat Genet. 2023;55:154‐164. 36564505 10.1038/s41588-022-01225-6 PMC10084891

[8] Zhang C , Zhang J , Xu FP , et al. Genomic landscape and immune microenvironment features of preinvasive and early invasive lung adenocarcinoma. J Thorac Oncol. 2019;14:1912‐1923. 31446140 10.1016/j.jtho.2019.07.031 PMC6986039

[9] Chen H , Carrot‐Zhang J , Zhao Y , et al. Genomic and immune profiling of pre‐invasive lung adenocarcinoma. Nat Commun. 2019;10:5472. 31784532 10.1038/s41467-019-13460-3 PMC6884501

[10] Dejima H , Hu X , Chen R , et al. Immune evolution from preneoplasia to invasive lung adenocarcinomas and underlying molecular features. Nat Commun. 2021;12:2722. 33976164 10.1038/s41467-021-22890-x PMC8113327

[11] Nie M , Yao K , Zhu X , et al. Evolutionary metabolic landscape from preneoplasia to invasive lung adenocarcinoma. Nat Commun. 2021;12:6479. 34759281 10.1038/s41467-021-26685-y PMC8580984

[12] Shang J , Jiang H , Zhao Y , et al. Differences of molecular events driving pathological and radiological progression of lung adenocarcinoma. EBioMedicine. 2023;94:104728. 37506543 10.1016/j.ebiom.2023.104728 PMC10406962

[13] Hu X , Fujimoto J , Ying L , et al. Multi‐region exome sequencing reveals genomic evolution from preneoplasia to lung adenocarcinoma. Nat Commun. 2019;10:2978. 31278276 10.1038/s41467-019-10877-8 PMC6611767

[14] Chen X , Wang D , Liu J , et al. Genomic alterations in biliary tract cancer predict prognosis and immunotherapy outcomes. J Immunother Cancer. 2021;9:e003214. 34795005 10.1136/jitc-2021-003214 PMC8603283

[15] Feng SH , Yang ST . The new 8th TNM staging system of lung cancer and its potential imaging interpretation pitfalls and limitations with CT image demonstrations. Diagn Interv Radiol. 2019;25:270‐279. 31295144 10.5152/dir.2019.18458 PMC6622436

[16] Wu T , Hu E , Xu S , et al. clusterProfiler 4.0: a universal enrichment tool for interpreting omics data. Innovation (Camb). 2021;2:100141. 34557778 10.1016/j.xinn.2021.100141 PMC8454663

[17] Sepulveda JL . Using R and Bioconductor in clinical genomics and transcriptomics. J Mol Diagn. 2020;22:3‐20. 31605800 10.1016/j.jmoldx.2019.08.006

[18] Wickham H . ggplot2: Elegant Graphics for Data Analysised. Springer Publishing Company, Incorporated; 2016.

[19] Cheng C , Amos CI . A refined use of mutations to guide immunotherapy decisions. Nature. 2022;612:639‐641. 36536222 10.1038/d41586-022-04448-z

[20] Mroz EA , Tward AD , Pickering CR , Myers JN , Ferris RL , Rocco JW . High intratumor genetic heterogeneity is related to worse outcome in patients with head and neck squamous cell carcinoma. Cancer. 2013;119:3034‐3042. 23696076 10.1002/cncr.28150 PMC3735618

[21] Melosky B , Kambartel K , Häntschel M , et al. Worldwide prevalence of epidermal growth factor receptor mutations in non‐small cell lung cancer: a meta‐analysis. Mol Diagn Ther. 2022;26:7‐18. 34813053 10.1007/s40291-021-00563-1 PMC8766385

[22] Wang S , Yu H , Gan Y , et al. Mining whole‐lung information by artificial intelligence for predicting EGFR genotype and targeted therapy response in lung cancer: a multicohort study. Lancet Digit Health. 2022;4:e309‐e319. 35341713 10.1016/S2589-7500(22)00024-3

[23] Zhou Q , Zhang XC , Chen ZH , et al. Relative abundance of EGFR mutations predicts benefit from gefitinib treatment for advanced non‐small‐cell lung cancer. J Clin Oncol. 2011;29:3316‐3321. 21788562 10.1200/JCO.2010.33.3757

[24] Shao Y , Zhong D . Detection and clinical significance of abundance of EGFR mutation. Zhongguo Fei ai Za Zhi. 2017;20:578‐583. 28855040 10.3779/j.issn.1009-3419.2017.08.11 PMC5973003

[25] Shi Y , Li J , Zhang S , et al. Molecular epidemiology of EGFR mutations in Asian patients with advanced non‐small‐cell lung cancer of adenocarcinoma histology ‐ mainland China subset analysis of the PIONEER study. PLoS One. 2015;10:e0143515. 26599344 10.1371/journal.pone.0143515 PMC4657882

[26] Zhou F , Guo H , Xia Y , et al. The changing treatment landscape of EGFR‐mutant non‐small‐cell lung cancer. Nat Rev Clin Oncol. 2025;22:95‐116. 39614090 10.1038/s41571-024-00971-2

[27] Campbell JD , Alexandrov A , Kim J , et al. Distinct patterns of somatic genome alterations in lung adenocarcinomas and squamous cell carcinomas. Nat Genet. 2016;48:607‐616. 27158780 10.1038/ng.3564 PMC4884143

[28] Qian J , Zhao S , Zou Y , et al. Genomic underpinnings of tumor behavior in in situ and early lung adenocarcinoma. Am J Respir Crit Care Med. 2020;201:697‐706. 31747302 10.1164/rccm.201902-0294OC PMC7068818

[29] Liao H , Zhang L , Lu S , Li W , Dong W . KIFC3 promotes proliferation, migration, and invasion in colorectal cancer via PI3K/AKT/mTOR signaling pathway. Front Genet. 2022;13:848926. 35812733 10.3389/fgene.2022.848926 PMC9257096

[30] Bahar ME , Kim HJ , Kim DR . Targeting the RAS/RAF/MAPK pathway for cancer therapy: from mechanism to clinical studies. Signal Transduct Target Ther. 2023;8:455. 38105263 10.1038/s41392-023-01705-z PMC10725898

[31] Sulzmaier FJ , Jean C , Schlaepfer DD . FAK in cancer: mechanistic findings and clinical applications. Nat Rev Cancer. 2014;14:598‐610. 25098269 10.1038/nrc3792 PMC4365862

[32] Wang L , Jia QZ , Chu Q , Zhu B . Targeting tumor microenvironment for non‐small cell lung cancer immunotherapy. Chin Med J Pulmon Crit Care Med. 2023;1:18‐29. 10.1016/j.pccm.2022.11.001 PMC11332857 39170874

[33] O'Neill HM , Lally JS , Galic S , et al. AMPK phosphorylation of ACC2 is required for skeletal muscle fatty acid oxidation and insulin sensitivity in mice. Diabetologia. 2014;57:1693‐1702. 24913514 10.1007/s00125-014-3273-1

[34] Macedo‐Silva C , Miranda‐Gonçalves V , Tavares NT , et al. Epigenetic regulation of TP53 is involved in prostate cancer radioresistance and DNA damage response signaling. Signal Transduct Target Ther. 2023;8:395. 37840069 10.1038/s41392-023-01639-6 PMC10577134

[35] Fagherazzi P , Stixová L , Bartova E . Specific TP53 mutations impair the recruitment of 53BP1 to DNA double‐strand breaks underlying the mechanism of radioresistance. Eur Biophys J. 2025. doi:10.1007/s00249-025-01774-8 PMC12678470 40659933

[36] Zhou G , Wang J , Zhao M , et al. Gain‐of‐function mutant p53 promotes cell growth and cancer cell metabolism via inhibition of AMPK activation. Mol Cell. 2014;54:960‐974. 24857548 10.1016/j.molcel.2014.04.024 PMC4067806

[37] Di Agostino S , Sorrentino G , Ingallina E , et al. YAP enhances the pro‐proliferative transcriptional activity of mutant p53 proteins. EMBO Rep. 2016;17:188‐201. 26691213 10.15252/embr.201540488 PMC5290815

[38] Liang P , Peng M , Tao J , et al. Development of a genome atlas for discriminating benign, preinvasive, and invasive lung nodules. MedComm (2020). 2024;5:e644. 39036344 10.1002/mco2.644 PMC11258453

[39] Xiong C , Ling H , Hao Q , Zhou X . Cuproptosis: p53‐regulated metabolic cell death? Cell Death Differ. 2023;30:876‐884. 36755067 10.1038/s41418-023-01125-0 PMC10070433

[40] Funk JS , Klimovich M , Drangenstein D , et al. Deep CRISPR mutagenesis characterizes the functional diversity of TP53 mutations. Nat Genet. 2025;57:140‐153. 39774325 10.1038/s41588-024-02039-4 PMC11735402

[41] Kimura E , Hayashi Y , Nakagawa K , et al. p53 deficiency in colon cancer cells promotes tumor progression through the modulation of Meflin in fibroblasts. Cancer Sci. 2025;116:1871‐1882. 40241262 10.1111/cas.70026 PMC12210049

[42] Alexandrov LB , Ju YS , Haase K , et al. Mutational signatures associated with tobacco smoking in human cancer. Science. 2016;354:618‐622. 27811275 10.1126/science.aag0299 PMC6141049

[43] Li Y , Li X , Li H , et al. Genomic characterisation of pulmonary subsolid nodules: mutational landscape and radiological features. Eur Respir J. 2020;55:1901409. 31699841 10.1183/13993003.01409-2019

[44] Fan Z , Zhang Q , Feng L , et al. Genomic landscape and prognosis of patients with TP53‐mutated non‐small cell lung cancer. Ann Transl Med. 2022;10:188. 35280362 10.21037/atm-22-412 PMC8908146

[45] Wu F , Fan J , He Y , et al. Single‐cell profiling of tumor heterogeneity and the microenvironment in advanced non‐small cell lung cancer. Nat Commun. 2021;12:2540. 33953163 10.1038/s41467-021-22801-0 PMC8100173

[46] Zhao J , Guo C , Ma Z , Liu H , Yang C , Li S . Identification of a novel gene expression signature associated with overall survival in patients with lung adenocarcinoma: a comprehensive analysis based on TCGA and GEO databases. Lung Cancer. 2020;149:90‐96. 33002836 10.1016/j.lungcan.2020.09.014

[47] Chen HY , Yu SL , Chen CH , et al. A five‐gene signature and clinical outcome in non‐small‐cell lung cancer. N Engl J Med. 2007;356:11‐20. 17202451 10.1056/NEJMoa060096

[48] Hong L , Patel S , Drusbosky LM , et al. Molecular landscape of ERBB2 alterations in 3000 advanced NSCLC patients. NPJ Precis Oncol. 2024;8:217. 39354054 10.1038/s41698-024-00720-9 PMC11445497

[49] Bi YY , Chen Q , Yang MY , Xing L , Jiang HL . Nanoparticles targeting mutant p53 overcome chemoresistance and tumor recurrence in non‐small cell lung cancer. Nat Commun. 2024;15:2759. 38553451 10.1038/s41467-024-47080-3 PMC10980692

