# Supplementary material for: Genomic signature driving preinvasive to invasive processes in stage I lung adenocarcinoma
Source: Int J Cancer. 2025 Dec 5;158(7):1975–88. doi: 10.1002/ijc.70282 (PMC12875189; doi:10.1002/ijc.70282)
Supplement: Supplementary file 3 — FIGURE S1. Gene mutation profiling of MIA and IA. FIGURE S2. Genomic events which may drive MIA to IA. FIGURE S3. Comparison of TMB, MATH score, and VA F scores between the IA and MIA cohorts associated with radiology, pathological and lesion diameter. FIGURE S4. Evaluation of the 11‐gene prognostic signature. FIGURE S5. Characterization of screened genes included in the risk stratification of the logistic regression model. [file IJC-158-1975-s001.pdf]

Genomic signature driving preinvasive to invasive processes in stage I lung adenocarcinoma

Biqin Mou, Yishan Duan, Jing Wang, Tiantian Li, Yuwei Huo, Xia Xiao, Conghui Cui, Zhujun Deng, Qiongxia Hu, Juan Jiang, Yiwei Liang, Sifen Lu, Xintong Tao, Kang Xie, Xinru Xiong, Niu Zhu, Liyun Bi, Faqiang Zhang, Weimin Li, Bojiang Chen

Table of Contents:

Supplementary Table 1. Gene list used in the 1021 gene panel testing (available in excel file).

Supplementary Table 2. Sequencing coverage and quality statistics of all samples used in this study (available in excel file).

Supplementary Figure 1. Gene mutation profiling of MIA and IA.

Supplementary Figure 2. Genomic events which may drive MIA to IA.

Supplementary Figure 3. Comparison of TMB, MATH score, and VAF scores between the IA and MIA cohorts associated with radiology, pathological and lesion diameter.

Supplementary Figure 4. Evaluation of the 11-gene prognostic signature.

Supplementary Figure 5. Characterization of screened genes included in the risk stratification of the logistic regression model.

Supplementary Tables:

Supplementary Table 1. Gene list used in the 1021 gene panel testing (available in excel file).

Supplementary Table 2. Sequencing coverage and quality statistics of all samples used in this study (available in excel file).

Supplementary Figures:

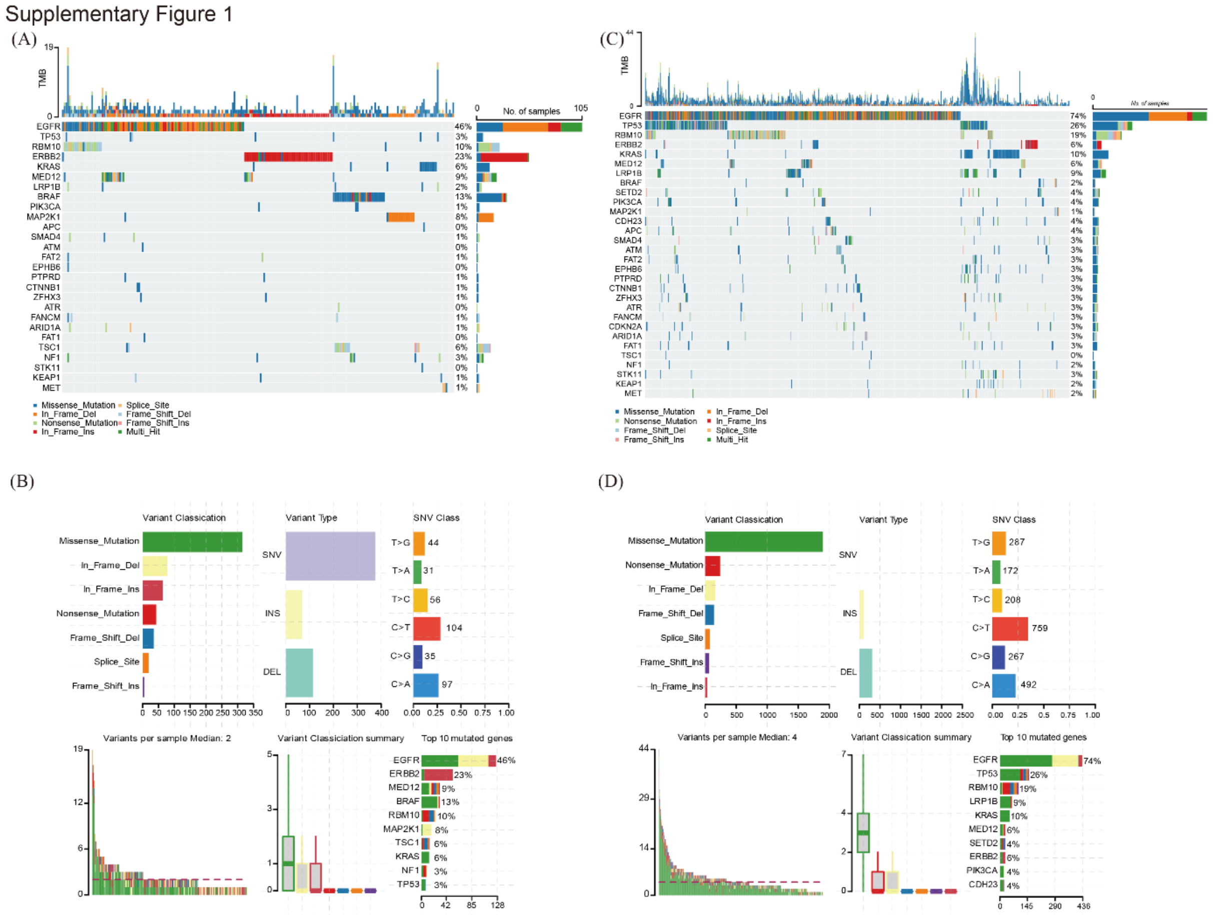

Supplementary Figure 1: Gene mutation profiling of MIA and IA. (A and B) Waterfall plots showing the frequency and types of mutations found in the TOP30 mutated genes in MIA (A) and IA (C) cohorts. (B and D) The distribution of mutation classifications of MIA (B) and IA (D).

Supplementary Figure 2

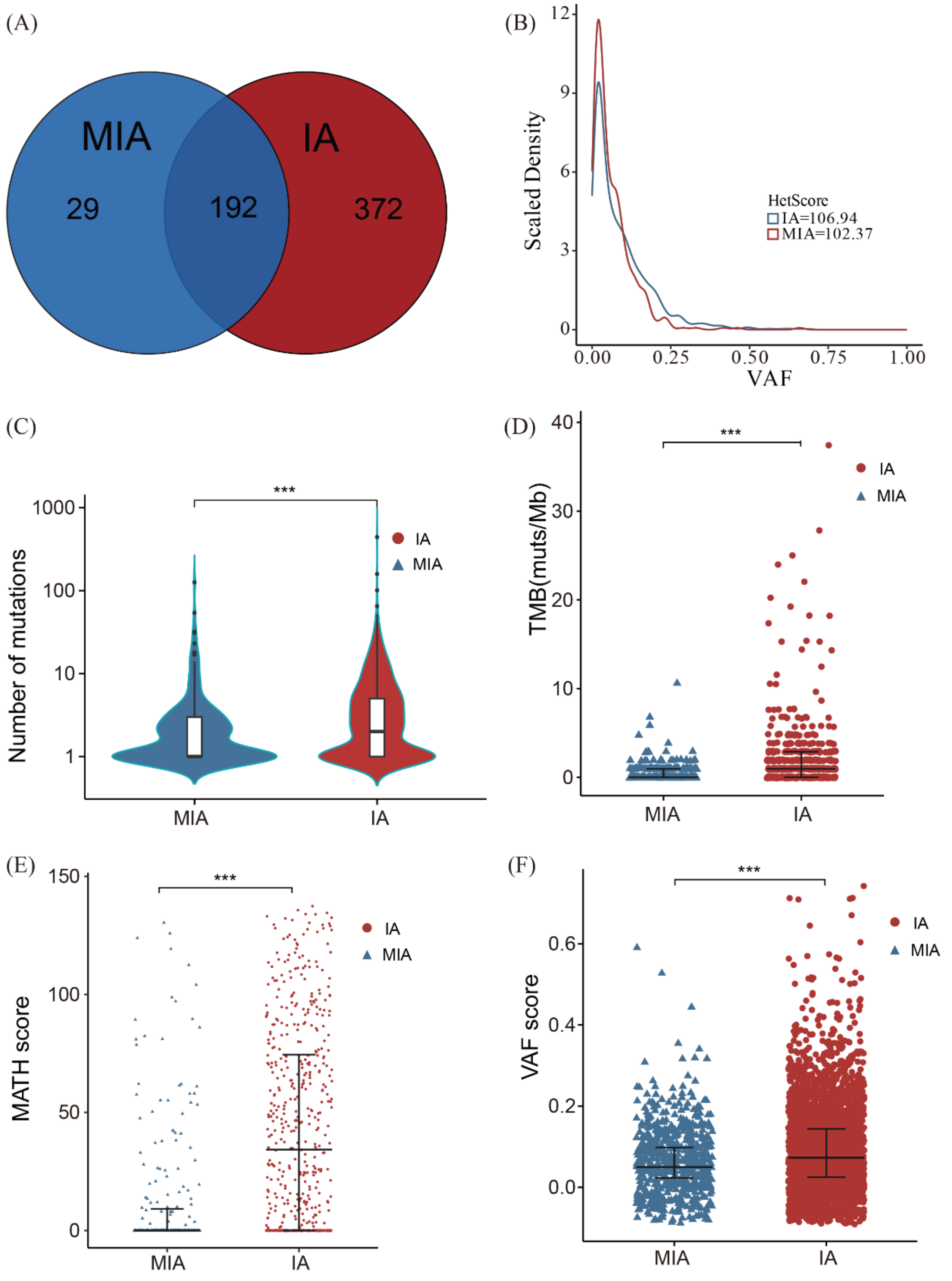

Supplementary Figure 2: Genomic events which may drive MIA to IA. (A) Overlap of 192 mutated genes between the two groups. (B) A density plot of the variant allele frequency of all SNVINDEL mutations according to histology and mutant-allele tumor heterogeneity scores is shown. (C-F) Comparison of TMB, MATH and VAF scores between MIA and IA. p values were adjusted by the false discovery rate (FDR). ***, $p < 0.001$.

Supplementary Figure 3

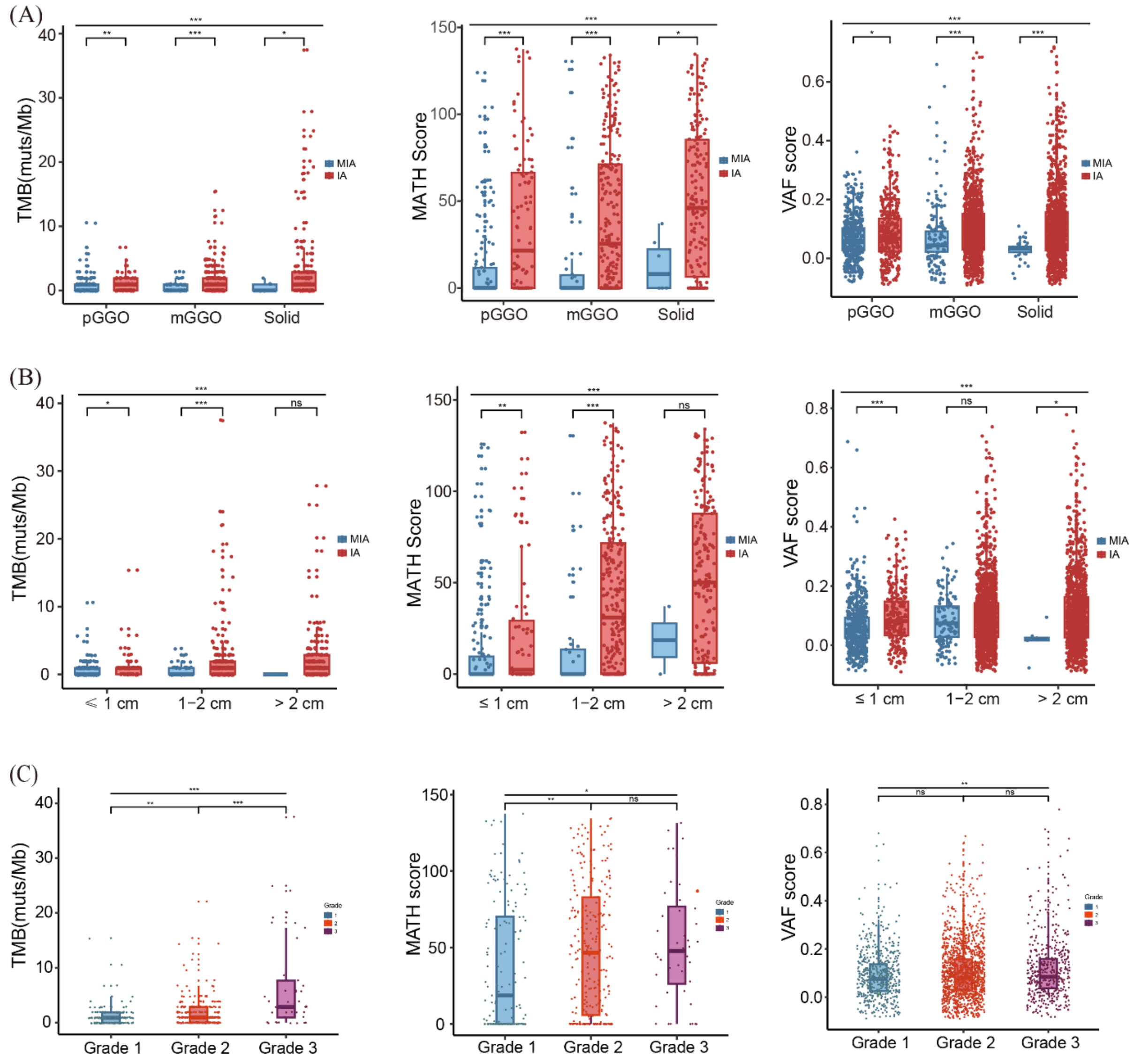

Supplementary Figure 3: Comparison of TMB, MATH score, and VAF scores between the IA and MIA cohorts associated with radiology, pathological and lesion diameter. (A) TMB, MATH score, and VAF score association with three radiology subtypes in MIA and IA. (B) TMB, MATH score, and VAF score association with lesion diameter in MIA and IA. (C) Comparison of TMB, MATH score, and VAF score in IA patients with different pathological progression stages (Grade 1 vs. Grade 2 vs. Grade 3). *, $p < 0.05$; **, $p < 0.01$; ***, $p < 0.001$, ^{ns}, not special.

Supplementary Figure 4

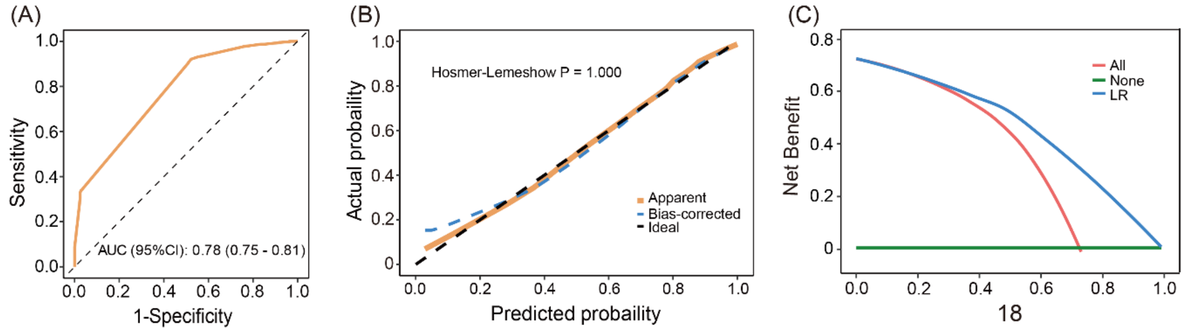

Supplementary Figure 4: Evaluation of the 11-gene prognostic signature. (A and B). Receiver operating characteristic (ROC) curve and calibration curve of the logistic regression model. AUC: area under the curve. (C) Decision curve analysis (DCA) of the logistic regression model. The horizontal line indicates that no patients develop IA, and the red oblique line indicates that patients develop IA.

Supplementary Figure 5

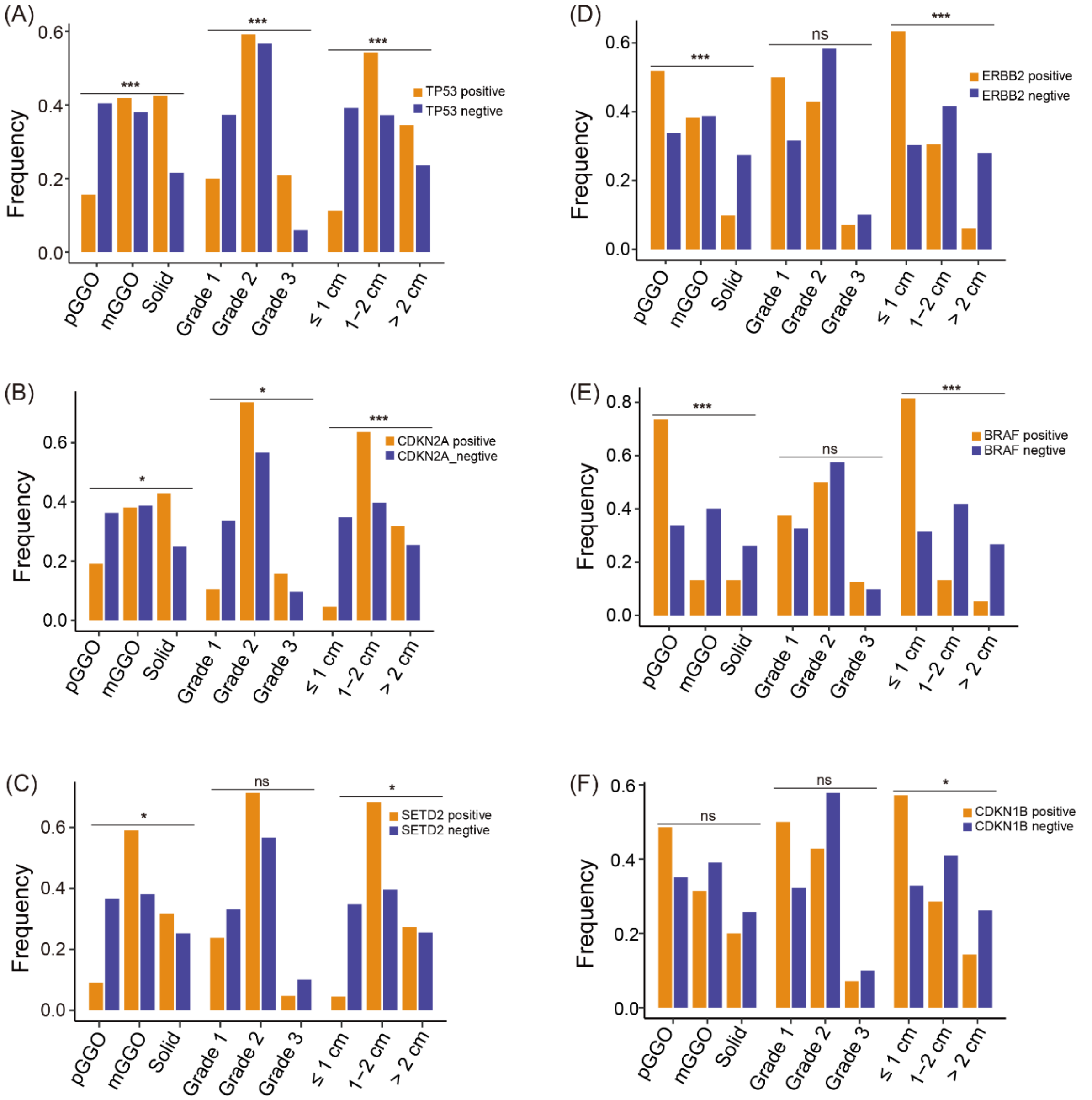

Supplementary Figure 5: Characterization of screened genes included in the risk stratification of the logistic regression model. (A-C) The association between *TP53*, *CDKN2A*, and *SETD2* mutations in the Gene3 set and radiological, pathological and lesion diameter of early lung adenocarcinoma. (D-F) The association between *ERBB2*, *BRAF*, and *CDKN1B* mutations in the Gene8 set and radiological, pathological and lesion diameter of early lung adenocarcinoma. *, $p < 0.05$; **, $p < 0.01$; ***, $p < 0.001$, ns, not special.
